# Supplementary material for: Disorder-assisted quantum transport in suboptimal decoherence regimes
Source: Sci Rep. 2016 Jan 4;6:18142. doi: 10.1038/srep18142 (PMC4698721; doi:10.1038/srep18142)
Supplement: Supplementary Information [file srep18142-s1.pdf]

Supplementary material: Disorder-assisted quantum transport in suboptimal decoherence regimes

Leonardo Novo*,[1] Masoud Mohseni,[2] and Yasser Omar[1,3]
[1] *Physics of Information Group, Instituto de Telecomunicações, P-1049-001 Lisbon, Portugal*
[2] *Google, Venice, CA 90291, USA*
[3] *CEMAPRE, ISEG, Universidade de Lisboa, P-1200-781 Lisbon, Portugal*
(Dated: 27th March 2015)

Dephasing-assisted transport without static disorder

The main text of this article focused on how the transport efficiency varies with the interplay between static disorder and pure dephasing, and also on how static disorder can assist transport in the suboptimal dephasing regime. Here, we focus on the zero-disorder scenario, and detail how dephasing assists transport in this regime. In Fig. S1 we show the dependence of the transport efficiency on the dephasing rate for both a binary tree and a hypercube with the same sizes considered in the main text (i.e. 5 generations and dimension 4, respectively), for different recombination rates $\Gamma = 0.01, 0.001$ and 0.0001. We see that, as dephasing tends to zero, the transport efficiency tends to the value $1/16$ for the binary tree (see Eq. 4 of the main text) and to the value $5/16$ for the hypercube (see Eq. 9 of the main text). The convergence to this value becomes more abrupt as the recombination rate lowers. For zero recombination rate, the transport efficiency should change abruptly from a fixed value at zero dephasing (obtained from Eq. 2 of the main text) to 1 at any finite value of dephasing, as predicted in [1]. For other examples of environment-assisted transport in the no-disorder case see also [2–4].

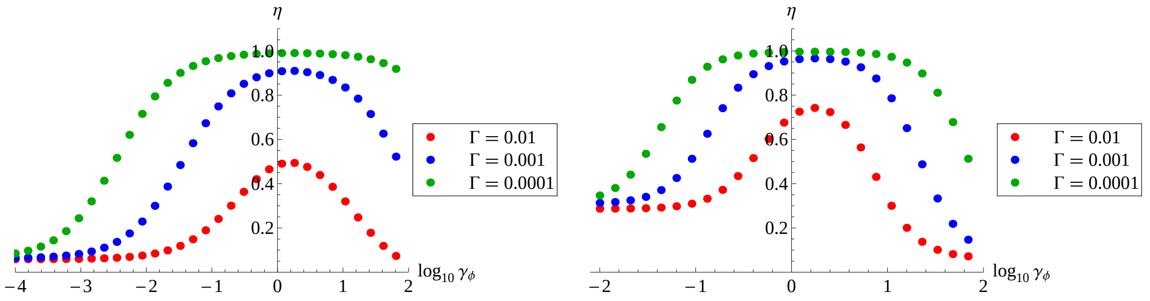

Fig. S 1: Transport efficiency on the binary tree (left panel) and hypercube (right panel) without static disorder ($\delta_\epsilon = 0$) as a function of the logarithm of the dephasing rate, for three different values of the recombination rate $\Gamma = 0.01, 0.001, 0.0001$. When dephasing tends to 0, we see the convergence of the transport efficiency to the value $1/16$ for the binary tree (see Eq. 4 of the main text) and to the value $5/16$ for the hypercube (see Eq. 9 of the main text). The optimality region for transport is reached when dephasing is ≈ 1, and this region is wider for smaller recombination rates, as expected.

[1] Caruso, F., Chin, A. W., Datta, A., Huelga, S. F. & Plenio, M. B. Highly efficient energy excitation transfer in light-harvesting complexes: The fundamental role of noise-assisted transport.

[2] Rebentrost, P., Mohseni, M., Kassal, I., Lloyd, S. & Aspuru-Guzik, A. Environment-assisted quantum transport. *New Journal of Physics* **11**, 033003 (2009).

[3] Kassal, I. & Aspuru-Guzik, A. Environment-assisted quantum transport in ordered systems. *New Journal of Physics* **14**, 053041 (2012).

[4] Wu, J., Silbey, R. J. & Cao, J. Generic mechanism of optimal energy transfer efficiency: A scaling theory of the mean first-passage time in exciton systems. *Phys. Rev. Lett.* **110**, 200402 (2013).
